# Dr. Bellamy's Case of Lacerated Urethra

**Published:** 1809-03

**Authors:** G. Bellamy

**Affiliations:** Hector, at Plymouth


### *To the Editors of the Medical and Physical Journal.*

Gentlemen,

WILL you do me the favour to insert the following Case of lacerated Urethra, from external violence? It is full of practical importance, and essential to be collated with what I formerly sent you, of disease in the same parts.

Robert Dainty, a seaman, of his Majesty's ship Glory, on the 20th of May, 1806, fell from the gangway, across the gunwhale of a launch along-side; he was much shook, in considerable pain, and a little faint; but walked with a little help to the sick birth. On examining the perinæum, there was no appearance of injury, except a trifling fullness, and scarce any pain to the touch; he so soon recovered as to be earnest to return to his labour, which I would not allow, fearing more serious consequences; a large compress with saturnine lotion was applied, and strict rest enjoined. My fears were too soon realised, for in about an hour afterwards, on attempting to make water, he fainted away, and not one drop passed, and yet he felt the bladder empty; a greater sense of fullness occupied the perinæum, but as yet no appearance of swelling or fluctuation. Anticipating great inflammation, he was immediately bled, ad ℔i. The cold lotion was assiduously applied, and an opiate given; two saline enemas were administered in the evening, which operated copiously; he had also a natural stool.

At 4 A. M. on 21st. v. s. ad ℔i. He was confined to bed, and the scrotum suspended. This day no fever, but repeated the enema, lotion, and kept him very low; only a little tea, and that as sparing as possible, to prevent secretion of urine. He lies quiet and half asleep; occasionally a very sharp pain for about a minute in an hour, about the membranous part of the urethra; extremely acute, as if the urine came so far and went back again. To the question, if he felt a sense of water trickling through? He said, Yes, something of that kind. A considerable rigour followed his fainting; very much like Sharp's case. The perinæum soon began to swell, and the scrotum likewise, followed by discolouration, and rapidly extending in every direction, so as to become this morning one livid mass; the whole scrotum and penis swelling, and as it were filling out together; so also around the perinæum and tuberosities of ischium, presenting a horrid appearance, like a part in a complete state of sphacelus; but it is chiefly the effect of extravasation and the blow, and the suppression of circulation, from the quantity of urine insinuated into the cellular membrane. The prepuce is puffed like a large phimosis, or as in extensive anasarca of the penis; the glans is quite hid, and in short, the whole of those parts are about six times their natural size. Perinæum very sensible to the touch, but no heat or tension; feels most like a bladder about half full; the region of the bladder is quite flat, and not sore, as if there was not a drop of water in it. From the first moment I suspected serious mischief, therefore acted as in a case of inflammation. It was now necessary to examine the state of the bladder, as he had not passed off any urine since 11, A. M. of 20th; the desire was continual, but every effort gave intense though not long pain in perinæum. Proceeded to ascertain the state of the bladder; at the first attempt it went very little beyond the membranous part of the urethra; felt some resistance; would not force, but withdrew the instrument; about two ounces of clear blood came off; a bad indication; shews internal rupture of blood vessels; some clots also came off. The introduction of the catheter did not give much pain, and at the second attempt it passed easily its whole length into the bladder, but not one drop of water came off; a little more blood, then fainting, rigour continuing, and the swelling fast advancing. I still directed my attention to the effects of inflammation; determined either that the bladder is burst, or the membranous part of the urethra lacerated. He had drank about a quart since the last micturition. Feels a sense of water passing, and constant filling of the perinæum and scrotum.

Got him into bed; had a stool in the evening rather involuntary; I emptied the rectum also by a stimulating enema, and seeing the importance of an empty bladder in case of inflammation, passed the catheter a third time; no force required or resistance made; no urine flowed; continued a very liberal application of the cold lotion, and in the the evening, when the circulation was restored from faintings, and having some fever, and a little fullness of pulse; bled him ad ℔i. Rep. enema, and gave opium, gr. 1.

Has had several slight rigors, and as his pulse is still full, bled again this morning, ad ℔j. at 4 A. M. since which he has looked pale and weak, pulse small, soft, and not quick.

Not a drop of water has passed, and bladder is quite flaccid; he feels about every hour the greatest desire to make water, and a few drops seem to pass through into the perinæum; and as this happens, so also the penis and scrotum distend, and also around the ischium, anus, &c. Except at these times of a few minutes sense of micturition, he lays pretty easy, and sleeps tolerably. What more can now be done, than first to obviate the danger of inflammation, which appears pretty well obviated at present; has no serious symptoms of fever, or pain in the belly; have less reason to fear rupture of the bladder, and increasing reason to believe that of the membranous part of the urethra; must wait, and persist against drink, although he begs and prays for it; as a great point will be to remove what fluid there is, and to prevent the accession of more, till nature can operate, if possible, some favourable change.

Through this day I gave up the apprehension of ruptured bladder, and became confirmed in that of the membranous part of the urethra, in which part, at night, the swelling was larger, fuller, and more tense, giving the feel of extravasated fluid; by lightness, most probably water, dispersed in the cellular membrane; the scrotum full the size of a small bullock's bladder; no fever, and tolerably easy, except at the time of sense of micturition. In the evening additional confirmation, if any required, for now the scrotum is so distended ~~that it~~ is not able to bear any more, and the region of the bladder above the pubis not a little distended, and become sensible to the touch, and his danger must rapidly increase, unless some evacuation be procured. Introducing two trocas in the depending and rather posterior part of the scrotum; a thin fluid oozed out, to the taste that of urine, and tinged with blood; secured the canulas, and got off before eight o'clock a full pint; of course, to the great relief of the patient, but yet no diminution of the size of the scrotum, because it filled up as fast as he feels the desire to micturate, but got off a pint and a half more to the evident great reduction of the bladder, and a little of the scrotum; about four ounces of blood mixed with the urine; one or two very slight chills in the night. Repeated the enema twice this day, and gave opium, gr. i. *vespere.* Enema feels comfortable, empties, even composes to sleep, though only of warm water.

May 22. To assist the perforation of the trocar, I evening, I made a few pretty deep incisions with a lancet in the scrotum and penis, and all oozed something and helped the general intention. In the middle watch last night, another pint and a half dropped off, so that at 4 A. M. there was a striking flaccidity of the whole, especially of the bladder, but the parts are equally discoloured, quite black, and only sensible to pain about the perinæum; slept tolerably well; forbears drink; has not had above a pint since hurt, to 8 this morning. The reduction of the swelling continues, and if I can keep him from fluids, and he is an obedient patient, may hope, if the perforations continue to ooze, for a total reduction of the swelling; he evidently has no fever, or other mark of inflammation. I cannot always keep him parching with thirst, but I have done I believe all that is justifiable at present; and if I have obviated general inflammation, may have now to apprehend only local inflammation, and consequent fistula in perinæo; but there is also danger of gangrene, communicated inflammation, and the great obscurity which all these parts, and the nature of their affection are wrapped in. I had an idea, if the plan of perforating the scrotum had failed, and the bladder had filled to excess, of perforating it; now to do that, the rectum is the least objectionable way; it would be a present indication of cure, for the water to flow that way, till nature, assisted by art, might restore the mischief in the canal. Repeat the enema twice a day, give opium gr. i. pro re nata; keep him low; keep the parts clean and cold by lot. saturn. At night looked rather sunk, and pulse weak; several rigors, and regular chattering of the teeth; scrotum and bladder kept well emptied, of course little or no oozing, only one pint the whole 24 hours; he cries out bitterly for a few moments, at each sense he has of the urine trickling from the bladder, which being an involuntary act, shews a paralyzed sphincter; swelling just kept at bay by oozing, and the absence of drink. Can take no food; great distress from thirst; lips a little black, pulse regular in time, skin without heat, perinæum yet very full, hard, and all round to the anus the whole is black, but sensible in every part to the touch; the most sensible part has always been the right groin, and the side of the scrotum touching that part. By frequent rigors, I apprehend matter is forming in the the perinæum; no hiccup nor mortification; thus may hope for formation of abscess, and fistulous opening for the urine, as the only probable chance of saving him. What if an artificial opening be made on purpose? but cannot tell the exact place; may do it below the wounded aperture, and communication with the cellular membrane; besides, why not hope to restore the urine to the regular canal? for if lacerated on the lower side, it does not follow when all swelling and inflammatory thickening and obstruction are gone, that the urine should not pass on through the regular canal. There has also been a glary discharge for some time after the accident, which I attributed to injury of the prostrate, but now in the form of matter, and is rather putrid; some confirmation of the hope that matter is forming; remember the state of such discharge from Sharpe, whose case was not much unlike it, and relief given to the narrowness produced by former inflammation, by the formation of abscess and fistula, and so might have lived, though a burthen, many years, if not for that unlucky secondary inflammation and gangrene; such will be the course here, either abscess or mortification, and to day 23d, by the severity of rigour, strong chattering of teeth, and more frequent recurrence, at least a dozen since 8 o'clock last night; hope for the formation of abscess in perinæo, which part continues very full and hard; not yet fluctuating, whereas all the rest remain lax, and very little larger than nature, and now able to draw back the prepuce. Matter oozes from the penis still, with a little blood; all the effect of the great bruise, giving hopes of matter forming there; injecting some warm water into the urethra, does not return, which confirms the opinion of the rupture of the membranes of the urethra, and that the water escapes into the cellular substance; therefore must be cautious how I repeat the injection.

Last evening he seemed to sink a little, very weak and pale, looks low to day. Allowed ℥ß. wine four times a day, just to keep up strength and tone, also a little soup; had yesterday about a gill of soup, and in the whole 24 hours about a pint of fluid; in fact, just as much as I think will ooze off, and keep the parts empty; some acid eructations, but no hiccup. Some fear of gangrene, because less pain in every respect, and particularly in that of water going through; does not cry out so much as he did. Apply large hot poultices to perinæum, and suspend scrotum well; continually apply hot fomentations to region of the bladder; last evening took an early hint by seeing a greater occurrence of rigor, and hopes of matter to leave off repellant and cold system, and began warm fomentations, which felt more comfortable, and necessary to encourage circulation in the parts so relaxed, and for fear of gangrene; shall also give a cordial in small form. Confect. aromatic gr. x. augendo de die, because most afraid of debility; thirst is highly distressing, but must persist against drink.

24. On the whole is worse, in great danger, scarce possible to live; the danger of gangrene has greatly increased; by noon, yesterday, the eructations amounted to hiccup, which has increased so as to shake and hurt him much; has had at least twenty fits of it; face sunk, eyes deadly; pulse though regular in time, is weak; skin cool, tongue brown and dry; lips and teeth somewhat black; no head-ach; and again, contrary to mortification, there are strong signs of inflammation and formation of matter; rigours much stronger, and longer, till 8 last night, since that slower and weaker, but yesterday very severe, by regular chattering of teeth; very distressing, yet he got a little sleep. I have continued strictly a large hot cataplasm and fomentation to the whole pubes, scrotum, &c. which ease and soothe; most of the orifices continue to ooze, but not equal to fluid taken, which was rather more indulged yesterday, on account of his cries for it, and to be allowed more freely; after a most pleasing circumstance, at 2 P. M. of passing the catheter, a very small one, pretty easily, when at the part supposed to be injured, and kept the point of it bearing on the upper side of the canal, for fear of entering the lacerated part, but it went completely in, and drew off a whole pint of water, to my great satisfaction and his delight; though by the uneasy position he almost fainted. Bladder did not feel, or appear to be full. I began to pass the catheter chiefly with a view, by gentle means, of ascertaining if any, and where, the obstruction might be, as also to induce a true passage by keeping it open, not unawares of the danger of exciting new inflammation. Scrotum had not enlarged, though felt harder, and believe it mostly to be by the water from the small syringe, injected to wash out matter and mucus, with a little blood from the penis; no water returned again when done last night, and scrotum seemed to get harder still; to be more backward in its use, especially if I can succeed occasionally passing the catheter, then may hope much, if gangrene does not advance; but loss of strength, faintness, hiccup, black tongue, &c. are strong and and dangerous signs; no delirium, but loss of spirits; feels dejected, and says, now is very ill; is very fretful. At night got worse; much pain in bottom of belly, no doubt from inflammation there; still directs chiefly to the right side of inguen, just above the pubes, where to day there is an evident swelling, very distinct from the bladder, and remaining after its evacuation, which was repeated this morning very happily, but not half a pint drawn off, yet the bladder seemed emptied by it, although so much more has been drank; this I account for by the greater oozing from the perinæum, where, on the left side, I made last night a deep incision, to ascertain if any matter; found none. Integuments very hard, by thickening; incision about half an inch long. I foresaw this advantage, if no matter was found, that by oozing of urine and blood, there would be a diminution of tension, hardness, fullness, and redness of that side; besides, having now access to the bladder, there appeared a diminution of all danger, but that of gangrene, and even of that, if I can keep up his strength without exciting inflammation of the bladder, or urethra; so must cautiously support and stimulate, to encourage maturation of the perinæum, by cataplasm and fotus. This may lessen a great cause of extensive irritation and mischief from effusiou of urine, if I am so happy to continue drawing it off, which I could not do at four this morning. At twelve, after being dressed, he expressed great uneasiuess, and sense of fulness of the bladder, but to external feeling not distended; greatest distress, tension, and pain is in the right inguen.

At 4, resistance to catheter, but not prudent to employ force. Gave opium gr. ij. last night, but scarce slept; and the distress and restlessness for drink so great, was obliged to increase the indulgence; in the last 24 hours has taken three half pints of tea; he looks forward to the hope of satisfying his thirst, as the greatest possible blessing.

At 9 this morning, after a few attempts with great caution, a very small catheter got in, but not the whole length, little further than neck of the bladder; yet so opened it, at least the obstructed part, that the urine flowed freely: now it may be that the urine is lodged without the bladder, and stopped by the lacerated part externally, where closed by inflammation, thickening, &c. He strained and vomited a little last night; not to day; and has kept down a gill of soup; also confect. aromatic gr. x. quart horis c. vin. ℥ß. with about four ounces of lemon juice, and as much wine besides. Seeing the great good of the last incision in perinæo, made a still more ample one on the other side, close down to the muscles; he slept a little to day, since water evacuated, and took a cup of tea; also last night a few ounces of gruel, and a tea spoonful of brandy in it.—No fœtor from the scrotum, and not vesicated or having any more putrescent appearance; rather wonderful with all the rigor, hiccup, &c. that a crisis has not taken place. To check hiccup, and stimulate gently, though it will excite water, yet given because it will at the same time cool, and be anti-putrescent, kali gr. v. in effervescence with acid wine, alternately every two hours, with confect. aromat. in vin. ℥j. singul. dos.

℞ Catap. fotus, &c.

25th. The poor fellow is closing his sufferings fast, they have been immense, but now less by the relief of delirium; he is so restless, that it is impossible to steady him; cannot now keep on any dressing, but have hitherto persisted in the use of cataplasm to perinæum and inguen, but on the latter the weight of it is too great; the part is so extremely sensible of pain, equal to touching the eye; kept tolerably composed, and had some sleep after drawing off about a pint of water yesterday.

At 4 A. M. took some tea at breakfast; felt refreshed, but the fever continued. Teeth quite black, tongue dry and brown; less hiccup all day; one or two slight rigors only, but state of parts worse, now in solution and perfect gangrene, but no fœtor; to day one spot, half dissolved, and breaking through at the bottom of the scrotum; it is altogether a most dreadful case of extreme anguish; so as to produce in a most firm mind, frequent restlessness and delirium, yet at times he is rational and patient.

I have succeeded, by great pains, in the application of the catheter, in drawing off at three different times, three half pints of urine; but the canal is full of interruptions, as if closed by inflammatory thickening, especially in the membranous part. We come now to a new view of the Case. The part he has always most complained of, was the right inguen, which yesterday appeared fallen, but by noon it became rather red and hot, more prominent, and extremely sensible to the touch; this redness, fullness, and degree of pointing by the pink colour of the centre, has gradually advanced.

There seems to be a general and deep thickening through the whole depth of the parietes of the abdomen, and I doubt not the explanation of all the rigors, internal pain, &c. is to be found in distinct inflammation of the coats of the the bladder; feel assured that if he could live long enough, should see suppuration of the bladder take place. But I anticipate that he is very soon, at all events, to finish the sum of his sufferings, as great as ever I beheld. We recollect how soon Sharp sunk under a nearly similar state; with the extraordinary combinations of inflammation and gangrene at the same time; there is excessive pain, heat, redness, rigors, disposition to suppuration, and actually local inflammation, whilst some parts are half putrid, and all along, since danger, a low fever; black teeth, dry brown tongue, and small pulse, cold skin, hiccup, discoloration; not all the effect of bruise and extravasation of blood and urine; it is truly a fit Case to be compared with Sharp's. In passing the catheter, not much pain expressed, which would be if the bladder was inflamed. Dissection would be highly explanatory and useful, as I had so much more pain in evacuating the bladder, and as thirst was his greatest distress, and had yesterday indulged him in various fluids, such as tea, succ. limon. and every two hours vin. ℥j. c. confect. aromatic and kali pp. as also with some soup, and a little wine occasionally. He often felt faint yesterday, and in the night, and once greatly so at 2 A. M. appeared to be going off; on the whole it appears vain, further to try any means of cure. Keep him as clean and quiet as possible, indulge his desire for drink, which has not been evacuated in proportion to what he has received; and as the scrotum was very tense, though not so large as formerly, yet I made several deep punctures in the scrotum last evening, from which there was a considerable oozing; this will allow the freer use of fluids, which appears to be his greatest consolation; and a mercy now to let him die as easy as I can.

26th. The poor fellow can scarce be said to be alive, and has not had a rational interval since 6 A. M. yesterday; the restlessness is immense, it is impossible to keep on any dressing. The eye became fixed about 4. Pulse gradually fell, scarce perceptible, body stretched out.

At 9, breathing laborious, and he was thought to be going off, but pulse got up again, and he seemed to be better the rest of the day; there was also more heat of the body, even of the feet than for two days past; which, with moisture of body, as is often felt before the last period, and which, to one unexperienced, might lead to an opinion that he was better, but it is only the last effort and labour of Nature.

27. He

27. He survived all yesterday till 7 P. M. when he breathed his last very quietly.

I proceeded to dissect. The first object was to ascertain the nature of the swelling of the groin; it was nothing more than extravasated urine, which the scrotum was not able to hold; it was evacuated upwards, only under the integuments a few ounces lodged there, and the cellular membrane was dissolved and corroded by it, and gangrene also; so that the extreme sensibility there, was the effect of inflammation of the integuments; muscles though thicker on that side, not decidedly inflamed, nor was any of the peritonæum attached to the bladder; found the bladder sunk deep in the pelvis, small, and hard, partly solid, and partly of that thickness produced by inflammation; its fundus externally red, not a drop of urine in it, firmly united, but not so strongly as Sharpe's, by adhesive layers, but yet very firmly to the peritonæum behind, and on its sides, and to the arch of the pubis. The next object was the state of the scrotum; integuments gangrened, the dartos was covered with fœtid matter, and nearly black, mixed with serum, urine, and blood; so also the same kind of matter beneath all the parts which were dissolved.

This layer of matter, supposed to be the cause of the repeated and severe rigors, but undoubtedly many of those rigors also attended as indicative of the great inflammation; for the integuments of the scrotum did not seem to have run into gangrene, as the effect of inflammation alone, but mostly, perhaps, the effect of extravasated blood, and disorganization from the fall. Tunica vaginalis thickened, and firmly united to septum scroti, but bodies of testes natural; so was also the cord. Now the great point is to refer to the perinæum; I dissected off the integuments cautiously, exposed in the anterior part (speaking as a body is usually placed for such dissection) of the membranous part of the urethra an aperture; decidedly not made by the knife; it has all the appearance of an old hole, about the size of a small oblong bean. I had previously introduced the catheter as far as it could go, but not into the bladder, even now after death; it appeared to be stopt by the neck of the bladder. Here was a full explanation of the causeand nature of his disease. In proceeding to take out the bladder, and dissecting the sides of the urethra, where it attaches to the arch of the pubis, found, on the right side, the knife unexpectedly to slip into a large cavity, exterior to the pelvis; when by taking off some of the integuments of perinæum, and towards wards the anus, saw a distended layer of the membranous part of the urethra, to the size of a small hen's egg; it was soft, and so putrid that it would not bear the knife, and being then accidentally divided, a few ounces of urine came out; so this part had formed a sac for urine, the parietes of which were formed above by the still remaining entire side of the urethra, till it came to the immediate laceration spoken of, which was rather on the left side; here the waters first escaped, since that, gradual solution of the inflamed sides of the urethra has gone on, so that at last inferior parietes of the sac were formed rather by the perinæum itself, which alone suspended the urine there lodged; this decay has been the work of the inflammation, and consequent gangrene: had it fortunately terminated in abscess at first, as was hoped for, fistula in perinæo might have saved him; but the contusion had been too great, and there was no indication for making an aperture in perinæo, for want of a place indicated; as also the state of the parts forbid it; moreover, till the last day, after the first inflammation, the urine had been evacuated by the catheter; in short, it appears, in my opinion, that nothing could have saved him.

Compare this case with that of poor Sharp; it is singular that I should have two such, so quick, and so highly important, interesting, and unfortunate in their consequences. In this case I would presume to fix the attention of the practical surgeon on this grand and leading particular; that when the urethra is divided, the passage of urine through such aperture, will not be a remote and secondary consequence, but a primary and immediate effect.

I am, &c.

G. BELLAMY, M. D.

*Hector, at Plymouth, Dec. 24. 1803.*

